# A Multi-Segmented Human Bioheat Model for Asymmetric High Temperature Environments

**DOI:** 10.3390/ijerph192215259

**Published:** 2022-11-18

**Authors:** Jing Geng, Yin Gu, Wenguo Weng, Ming Fu, Shifei Shen, Rui Zhou

**Affiliations:** 1 Institute of Public Safety Research, Department of Engineering Physics, Tsinghua University, Beijing 100084, China; 2 Beijing Key Laboratory of City Integrated Emergency Response Science, Tsinghua University, Beijing 100084, China; 3 Hefei Institute for Public Safety Research, Tsinghua University, Hefei 230601, China; 4 Anhui Province Key Laboratory of Human Safety, Hefei 230601, China

**Keywords:** asymmetric high-temperature environments, human bioheat model, human physiological responses

## Abstract

In workplaces such as steel, power grids, and construction, firefighters and other workers often encounter non-uniform high-temperature environments, which significantly increase the risk of local heat stress and local heat discomfort for the workers. In this paper, a multi-segment human bioheat model is developed to predict the human thermal response in asymmetric high-temperature environments by considering the sensitivity of the modeling to angular changes in skin temperature and the effects of high temperatures on human thermoregulatory and physiological responses simultaneously. The extended model for asymmetric high-temperature environments is validated with the current model results and experimental data. The result shows that the extended model predicts the human skin temperature more accurately. Under non-uniform high-temperature conditions, the local skin temperature predictions are highly consistent with the experimental data, with a maximum difference of 2 °C. In summary, the proposed model can accurately predict the temperature of the human core and skin layers. It has the potential to estimate human physiological and thermoregulatory responses under uniform and non-uniform high-temperature environments, providing technical support for local heat stress and local thermal discomfort protection.

## 1. Introduction

Non-uniform high-temperature environments are frequently encountered in the workplace of firefighters and other personnel working in the steel, power grid, and construction industry. The risk of local heat stress and local thermal discomfort for workers increases significantly due to exposure to unilateral fire or unilateral intense heat. Without appropriate protection measures, it would lead to irreparable local burn injuries or even life-threatening injuries. Knowledge of human physiological and thermoregulatory responses to non-uniform high-temperature environments is important for assessing physiological performance, comfort, and safety. As a result, developing models of human thermophysiological responses appropriate for extremely non-uniform high-temperature environments is critical.

Mathematical modeling of thermal physiological responses is one of the most effective and valuable tools for simulating human physiological and thermoregulatory responses [1]. Some cutting-edge academic organizations and groups conducted a series of related studies and achieved numerous results, including greatly preventing the heat stress response of the human body and improving the thermal comfort of the human body in high-temperature environments. Firstly, the human bioheat model applicable to uniform thermal environments was developed and widely used in the prediction of human physiological and thermoregulatory response, risk analysis of heat stress, and the prediction of survival time [2]. The first mathematical model was proposed by Pennes [3] and Weinbaum and Jiji [4]. Subsequently, the model development went into two stages from the two-node model developed by Gagge [5], to the multi-node and multi-element model initially proposed by Stolwijk [6,7], and Wissler [8]. One of the most classic models was developed by Stolwijk, which divided the human body into six parts, each of which were divided into four nodes: core, fat, muscle, and skin. On this basis, many multi-segment multi-layer human bioheat models are modified and improved by Huizenga and Hui [9], Fiala [10], Tanabe and Kobayashi [11,12], Salloum [13], Ferreira and Yanagihara [14], Zolfaghari and Maerefat [15], Karaki [16], Weng [17], Yang [18], Xie and Niu [19], Takahashi [20], Marian [21], and Wu [22].

However, the human bioheat models described above mainly focus on the heat exchange processes between the human body and the environments in uniform hot or cold environments. These models could predict the mean skin temperature but are not suitable for the local skin temperature prediction of the human body. Therefore, it is necessary to establish the non-uniform bioheat model to predict the local skin temperature by considering the angular changes between the body surface and the heat source. It will be used for local thermal sensation assessment, but also prevent damage to the human body caused by local overheating. Recently, a few multi-segmental bioheat simulation models interned the simulation of human thermal response in spatially non-uniform thermal environments [23,24,25,26,27,28,29,30,31] by dividing the skin nodes with different angular identical parts. The asymmetric high-temperature environment studied in this paper, as a kind of non-uniform environment, not only affects the heat exchange between the human body and heat source but also is often accompanied by significant differences in the human thermal response under high-temperature environments. The above models for asymmetric environments primarily predict human thermophysiological responses in the normal temperature range, the upper limit of which does not exceed 40 °C. Firefighters and others who work in the steel, power grid, and construction industries work in a typical non-uniform thermal environment. The effects of high temperature on metabolic rate and the cardiovascular system, as well as the role of heat-induced dehydration on sweating and other thermophysiological regulatory reactions often occur. Long-term exposure will seriously harm health and even cause irreversible damage. To date, a human bioheat model for non-uniform high-temperature environments used to study human thermal response is not published.

In this paper, we strive to present a multi-segment human bioheat model based on Salloum’s work for human thermal response prediction in asymmetric high-temperature environments. First, Salloum’s model [13] is extended to non-uniform environments by dividing the skin nodes into four angular identical parts. Next, the special human thermal responses caused by high temperatures are introduced, which extend the modified model to asymmetric high-temperature environments. Finally, the model proposed in this paper is validated and evaluated with published experimental data in both uniform and non-uniform high-temperature environments.

## 2. Model Development

In this section, we first develop a human bioheat model for non-uniform environments based on Salloum’s work and then extend it to consider the effect of high temperature on the human physiological and thermoregulatory response [17,32,33]. In general, the extended human bioheat model simulates the thermal response through two interacting systems: the passive system and the controlled active system. The passive system includes the energy balance integral equation and the circulation system for modeling the heat transfer that occurs. To obtain the local skin temperature in different regions of each segment, this paper expands the number of skin nodes in each segment by direction division to improve the passive system. The active system is used to model the thermoregulatory response in the human body controlled by the hypothalamus. This paper improves the model by extending physiological responses, such as metabolic rate changes, cardiovascular system changes, and sweating rates, induced by high temperatures.

### 2.1. Modeling Asymmetric Passive Systems

According to Salloum’s model, in the passive system, the human body was subdivided into 15 cylindrical segments: head, chest, abdomen, upper arms, forearms, hands, thighs, calves, and feet. Each segment contains a core node, a vein node, an artery node, and a skin node. Heat transfer between different segments is accomplished by the blood flow between adjacent body segments, including arterial blood from the heart to various body segments and back to the heart with venous blood. The heat transfer process in the same segment includes conduction, convection, and perfusion between different nodes. Blood perfusion included from the arterial node to the core, and from the core node to the skin node. Convection occurs between the arterial and venous blood flow and the core layer, and conduction occurs between core layer nodes and adjacent skin nodes.

**Figure 1:**
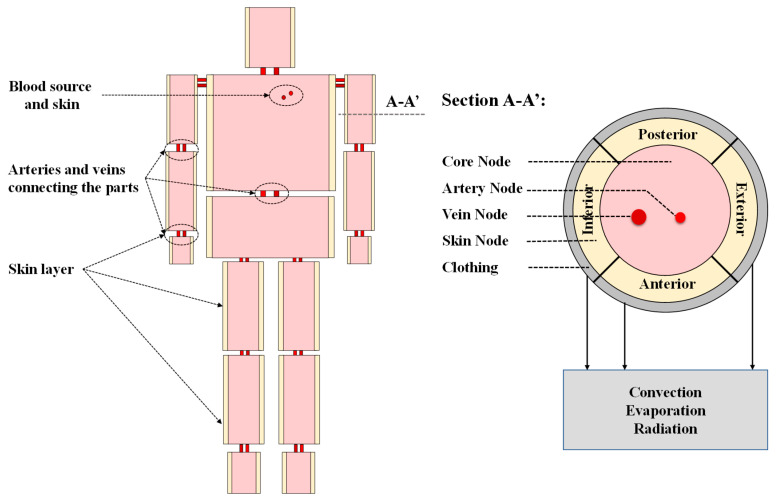
Schematic diagram of the multi-segment multi-node model.

However, given the actual operating scenarios of firefighters and workers in the steel, outdoor power grid, and construction industries, they are often exposed to intense heat on one or more sides. Therefore, the skin node was decomposed into four equal sectors (anterior, exterior, posterior, and inferior) in the proposed model by referring to the model of Kubaha [34]. Thence, the asymmetric human model is developed, which includes 15 segments, each including a core node, a vein node, an artery node, and four skin nodes, for a total of 105 nodes, as shown in Figure 1. The detailed parameters of each segment are shown in Table 1.

**Figure 2:**
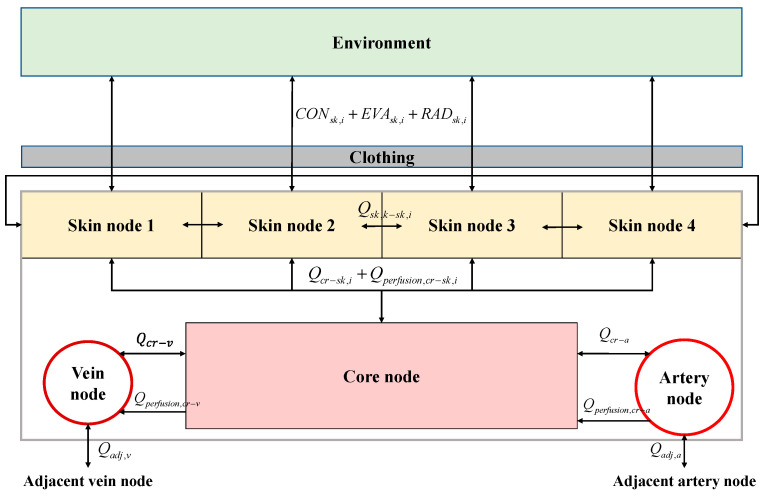
Schematic diagram of heat transfer between the core, artery, vein, and four skin nodes.

For each segment, the schematic diagram of heat transfer between the core, artery, vein, and four skin nodes is shown in Figure 2. The energy balance equations for the seven nodes after modification of Salloum’s energy balance equation [13] are as follows.

For the core node

(1) $$C_{cr}\frac{\partial T_{cr}}{\partial t}=(M_{cr}-W_{cr})-\sum_{i=1}^{4}Q_{cr-sk,i}-\sum_{i=1}^{4}Q_{perfusion,cr-sk,i}-Q_{perfusion,a-cr}-Q_{cr-a}-Q_{cr-v}-RES$$

where *t* is the time. $C_{cr}$ and $T_{cr}$ are the heat capacitance and the temperature of the core node, respectively. *M_cr_* is the basal metabolic rate of the core. *W* is the mechanical work made in the core. $Q_{perfusion,a-cr}=\dot{m_{cr}}c_{bl}(T_{bl,a}-T_{cr})$ is the perfusion heat transfer of the core, which is associated with the blood perfusion in the core $\dot{m}$ *_cr_* and the temperature of the core node $T_{cr}$ and artery node $T_{bl,a}$. *c_bl_* is blood-specific heat, usually taking the value of 4000 J/kg. $Q_{cr-a}=\sum h_{a}A_{a}(T_{cr}-T_{bl,a})$ and $Q_{cr-v}=\sum h_{v}A_{v}(T_{cr}-T_{bl,v})$ are the convection heat transfer between the core, and the artery, and vein, respectively; *h_a_* and *h_v_* are heat convection coefficients, which can be calculated by referring to the human circulatory system model of Salloum et al. [13]. In addition, for the core layer of the chest, there is also respiratory heat loss *RES* = 0.0014*M_cr_*(34 − *T_air_*) + 0.0173*M_cr_*(5.87 − *P_air_*), which is generated by convection and evaporation [35].

Particularly, $\sum_{i=1}^{4}Q_{cr-sk,i}=\sum_{i=1}^{4}\left\{K_{cr-sk,i}(T_{sk,i}-T_{cr})\right\}$ represents the conduction heat transfer between skin node *i* and the corresponding core node. $K_{cr-sk,i}$ is the thermal conductance between the skin node *i* and to the core node. $\sum_{i=1}^{4}Q_{perfusion,sk,i-cr}=\sum_{i=1}^{4}\left\{{\dot{m}}_{sk,i}\cdot c_{bl}\cdot \left(T_{sk,i}-T_{cr}\right)\right\}$ represents the blood perfusion volume of skin layer *i* to the core, which is associated with the skin perfusion blood flow rate ${\dot{m}}_{sk,i}$ and the temperature of the skin node *i* $T_{sk,i}$ and core node $T_{cr}$. The effect of non-uniform environments on the blood perfusion rate of the skin node *i* is evaluated by the parameter $\delta_{i}$, that is ${\dot{m}}_{sk,i}={\dot{m}}_{sk,perfusion}\left(1+\delta_{i}/\sum {}_{i}\max(0,\delta_{i})\right)$, where $\delta_{i}=T_{sk,i}-\left(\sum_{i=1}^{4}T_{sk,i}\right)/4$.

For the artery and vein node

(2) $$C_{bl,a}\frac{dT_{bl,a}}{dt}=Q_{cr-a}+Q_{adj,a}$$

(3) $$C_{bl,v}\frac{dT_{bl,v}}{dt}=Q_{cr-v}+Q_{perfusion,cr-v}+Q_{adj,v}$$

where $C_{bl,a}$ and $C_{bl,a}$ are the heat capacitance of the artery and vein node, respectively. $Q_{perfusion,cr-v}\dot{={\dot{m}}_{cr}}c_{\mathrm{bl}}(T_{cr}-T_{bl,v})$ is the part considered by perfusion of heat transfer between the core and vein node. $Q_{adj,a}=\dot{{\dot{m}}_{a}}c_{\mathrm{bl}}(T_{bl,a,adj}-T_{bl,a})$ and $Q_{adj,v}=\dot{{\dot{m}}_{v}}c_{\mathrm{bl}}(T_{bl,v,adj}-T_{bl,v})$ denote the heat exchanges caused by the blood entering the artery node, and the same process taking place in the vein node, respectively. ${\dot{m}}_{a}$ and ${\dot{m}}_{v}$ are the correspondence mass flow rates.

Finally, the energy balance equation for the four skin nodes after modification of Salloum’s energy balance equation is given by

(4) $$C_{sk}\frac{\partial T_{sk,i}}{\partial t}=K_{sk,i}L_{n}th_{sk,i}\frac{1}{r^{2}}\frac{\partial^{2}T_{sk,i}}{\partial \theta^{2}}+M_{sk,i}+Q_{cr-sk,i}+Q_{perfusion,}{}_{cr-sk,i}-CON_{sk,i}-EVA_{sk,i}-RAD_{sk,i}$$

where $C_{sk}$ is the heat capacitance of the skin node *i* (*i* = 1–4). The first term on the right-hand side of the equation indicates the conduction heat exchange between each skin node in the same segment, where, $K_{sk,i}$ denotes the skin thermal conductivity, $L_{n}$ denotes the segment length, $th_{sk,i}$ denotes the thickness of the skin layer in the segment, *r* is the radius of the skin layer, and θ is the corresponding angular coordinate of the skin node. $M_{sk,i}$ is the metabolic rate of the skin node and is equal to the total metabolic rate of the segment divided by the number of the skin node.

$CON_{sk,i}$, $EVA_{sk,i}$, and $RAD_{sk,i}$ are the heat exchanges between the skin node *i* and the external environment caused by convection, evaporation, and radiation, respectively.

For the exposed segments of the skin, we have

(5) $$CON_{sk,i}=A_{sk,i}h_{c}(T_{sk,i}-T_{air,i})$$

(6) $$EVA_{sk,i}=A_{sk,i}h_{e}(P_{sk,i}-P_{air,i})$$

(7) $$RAD_{sk,i}=RAD_{sk,i,1}+RAD_{sk,i,2}$$

where $RAD_{sk,i,1}=A_{sk,i}h_{r}(T_{sk,i}-T_{mrt})$ is the radiation heat transfer when there is no intense heat on one or more sides, and $T_{mrt}$ is the mean ambient radiation temperature. When there is intense heat on one side *i*, an additional $RAD_{sk,i,2}=\sigma \cdot \varepsilon \cdot F_{vf}\cdot \left[{\left(T_{sk,i}+273.15\right)}^{4}-{\left(T_{r}+273.15\right)}^{4}\right]$ needs to be introduced to represent the radiative heat transfer between the strong radiation source and the skin node *i* of the corresponding segment. $T_{r}$ is the temperature of an intense heat source. $F_{vf}$ is the view factor, which can be calculated based on the human body location and the radiation source distribution. The case will be configured according to the actual conditions and calculated using the additivity of the view factor [10].

For clothed body segments, the simplified clothing model described by Yang et al. [18], which was proven to be reasonably accurate [18,22], was adopted in this study.

### 2.2. Modeling of Active System

The thermoregulatory control system consists of four activities: skin vasoconstriction, vasodilation, shivering, and sweating. For vasomotor, the same governing equations of Salloum et al. [13] were adopted in this model. In particular, the mean skin temperature $T_{sk,mean}$ and skin blood perfusion rate ${\dot{m}}_{sk,perfusion}$ of the corresponding segment are given by [26].

(8) $$T_{sk,mean}=\sum_{i=1}^{4}\frac{T_{sk,i}}{4}$$

(9) $${\dot{m}}_{sk,perfusion}=\sum_{i=1}^{4}{\dot{m}}_{sk,i}$$

In addition, the sweating and the shivering thermoregulatory function follows the JOS model of Tanabe [11] and Kobayashi [12], and will not be presented here. The distribution coefficients used in this paper are shown in Table 2. *T_sk_*_0_ is the set point temperature of the corresponding segment, and the thermal reference core temperature of the head is set as 36.9 °C [11]. *α_sk_* is the skin sensitivity. *α_sw_*, and *α_sh_* are the distribution coefficients of sweating and shivering, respectively.

### 2.3. Extension to High-Temperature Environments

The thermal response of the human body is affected by parameters such as ambient temperature, humidity, and wind speed. The human physiological response will change as the temperature increases when other conditions remain unchanged. In the working environment of firefighters and others working in the steel, power grid, and construction industries, the temperature is usually higher than 40 °C. The external environment mainly transmits heat to the human body through convection and radiation. Therefore, to ensure that the core temperature of the human body is within the normal range, the thermoregulatory system of the human body responds differently compared with normal environments. It leads to several significant differences, such as the effects of high temperatures on metabolic rate, cardiovascular system, and the role of heat-induced dehydration on sweating and skin vasodilation. Therefore, the extended non-uniform high-temperature environment model should consider the above specific thermal response of high temperature to the human body.

#### 2.3.1. Effects of High Temperatures on Metabolic Rate

In high-temperature environments, heat stress causes an increase in the intensity of the metabolism, which provides energy for thermoregulatory activities. According to Weng et al. [17], the metabolic rate increases by 10–13% for every 1 °C increase in body temperature, when the environment temperature is greater than 39 °C. This process should be considered in the extended model. The relationship between the proportional rising in human metabolic rate *M* and the increase in body temperature $T_{cr}$ can be expressed as [17]

(10) $$M=\left\{\begin{array}{l} M_{0}\\ (1+0.13(T_{cr}-39))M_{0} \end{array}\right.\begin{array}{l} T_{a}<39\;{}^{\circ}C\\ T_{a}\geq 39\;{}^{\circ}C \end{array}$$

where $M_{0}$ is the metabolic rate at room temperature.

#### 2.3.2. Effects of High Temperatures on Cardiovascular System

When the ambient temperature rises, the heart activity will increase to meet the needs of the human body for heat dissipation and oxygen supply. Wenzel et al. [36] indicated a slow increase in heart rate over time in a hot environment. The increase in heart rate ΔHR can be calculated by [33,36]

(11) ΔHR=4.75×D

where $D=100\times \int_{0}^{t}Sweat\times dt/74.30\left(\%\right)$ is the percentage of cumulative water lost by the body.

#### 2.3.3. Role of Heat-Induced Dehydration on Sweating

According to Zhao et al. [33], the percentage of water loss to the total body weight decreases sweating sensitivity and increases sweating threshold temperature to reduce sweating. Therefore, in the modified model, we considered the role of calculated water loss *D* in the sweating regulation by introducing the modified equation for sweating thermoregulatory function proposed by Montain et al. [37].

(12) $$Sweet(i)=(0.2898-0.068D)(T_{head}-T_{head,set}-0.06D)+0.03364\left(Wrms-Clds\right)$$

where $T_{head}$ is the head core temperature, $T_{head,set}=37\;{}^{\circ}C$ is the set-point temperature, and *Wrms* and *Clds* are integrated signals from skin thermoreceptors. That is to say, each 1% increase in cumulative dehydration resulted in a 0.06 °C increase in the hypothalamic thermoregulatory point threshold, while resulting in a 0.068 kg/(h·°C) decrease in the sweat sensitivity of sweating.

## 3. Results and Discussion

In this paper, MATLAB (Math Works, Inc.) is used to solve the multi-segment human bioheat model in a non-uniform environment. Firstly, the simulated core temperature is validated using the existing experimental data and the simulation results in a uniform high-temperature environment. In an asymmetric high-temperature environment, the improved model was validated using the skin temperature data of different body segments reported by Fanger [38] in a non-uniform high-temperature environment.

### 3.1. Comparison with Uniform High-Temperature Environments

**Figure 3:**
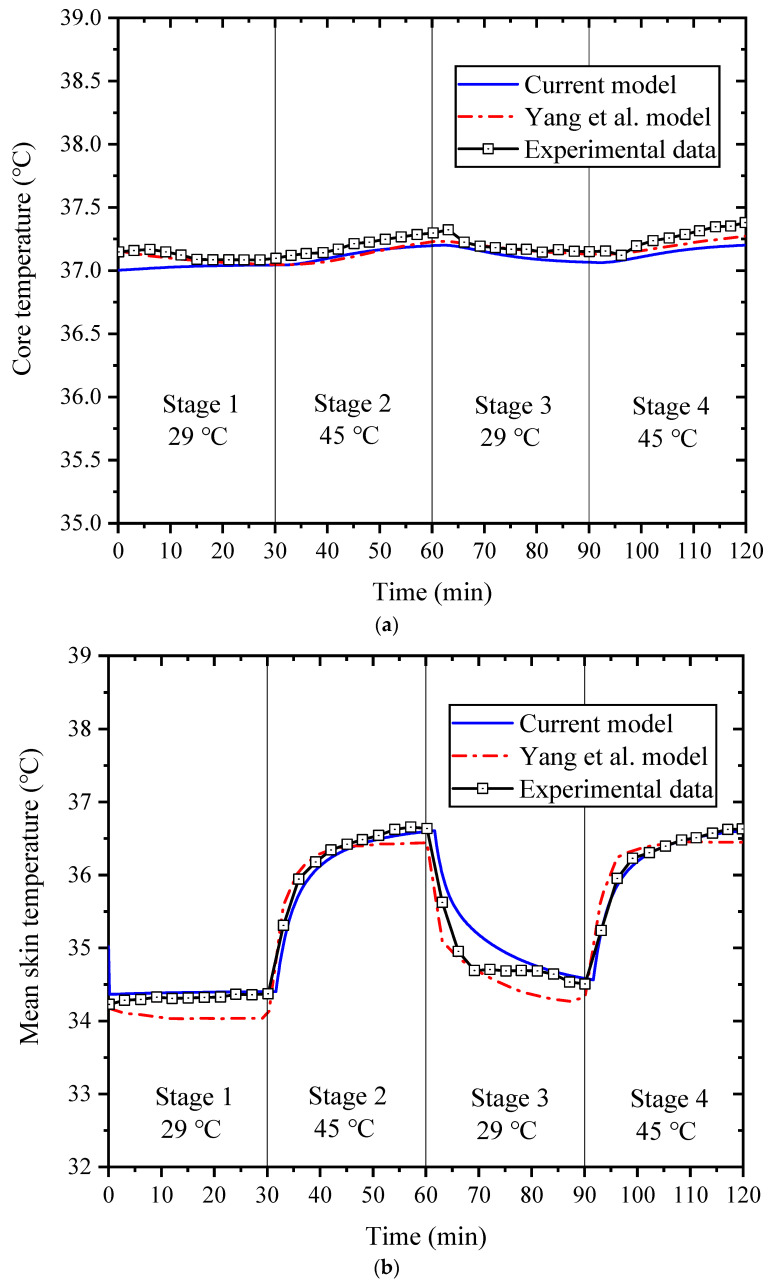
Comparison of core temperature (**a**) and mean skin temperature (**b**) between the experimental data and simulated results of Yang et al. [18] and the simulated results calculated with the current model.

The core and mean skin temperatures simulated by the extended model were validated by comparing them with published experimental data and simulation results by Yang et al. [18]. In the experiments, five healthy males (wearing only shorts) were exposed to four conditions: first for 30 min in a neutral environment (29 °C; Stage 1), followed by 30 min in a hot environment (45 °C; Stage 2), and then back to the neutral environment (29 °C; Stage 3) for 30 min, finally in the hot environment (45 °C; Stage 4) for 30 min. During the experiments, the core and skin temperatures of each subject were measured. Figure 3 shows the comparison of (a) the core temperature and (b) the mean skin temperature between the experimental data and simulated results of Yang et al. [18] and the simulated results calculated with the current model. The relative errors between the predicted results of the two models and the experimental data are shown in Table 3. It is clear that the simulation results are in good agreement with the experimental data, and the maximum relative error is only ±1.62%. The core temperature predictions were within ±0.48% of relative error to the experimental data, which is comparable to the agreement shown by the model of Yang et al. [18]. The temperature prediction of the mean skin temperature was very close to the experimental data, especially under high-temperature conditions (stage 2 and stage 4), with a relative error of ±0.67%, which was significantly better than the model of Yang et al. [18].

This may be because the current model takes into account the effect of high temperature on metabolic rate as well as heart rate, which results in higher human metabolic rate and skin blood flow rate in the extended model in a hot environment than in the Yang et al. model. In addition, the role of heat-induced dehydration on sweating is introduced in the current model and well represents the sweating effect of the human body in a hot environment. Therefore, the validation results indicate that the extended model can accurately predict the core temperature and skin temperature in uniform high-temperature environments.

### 3.2. Comparison with Asymmetric High-Temperature Environments

A set of asymmetric radiation experiments was reported by Fanger et al. [38] in 1985. In the experiment, 16 college students (8 males and 8 females, standard KSU uniforms) were at a distance of 0.5 m from the warming wall. During the first hour of the experiment, the warming wall was not heated (23 °C). Subsequently, the warm wall temperature was adjusted every half hour (32.6 °C, 42 °C, 51.6 °C, 61.1 °C, and 70.1 °C), while the ambient air temperature was down (21.9 °C, 20.7 °C, 19.3 °C, 17.9 °C, and 16.7 °C) to keep the operating temperature stable.

**Figure 4:**
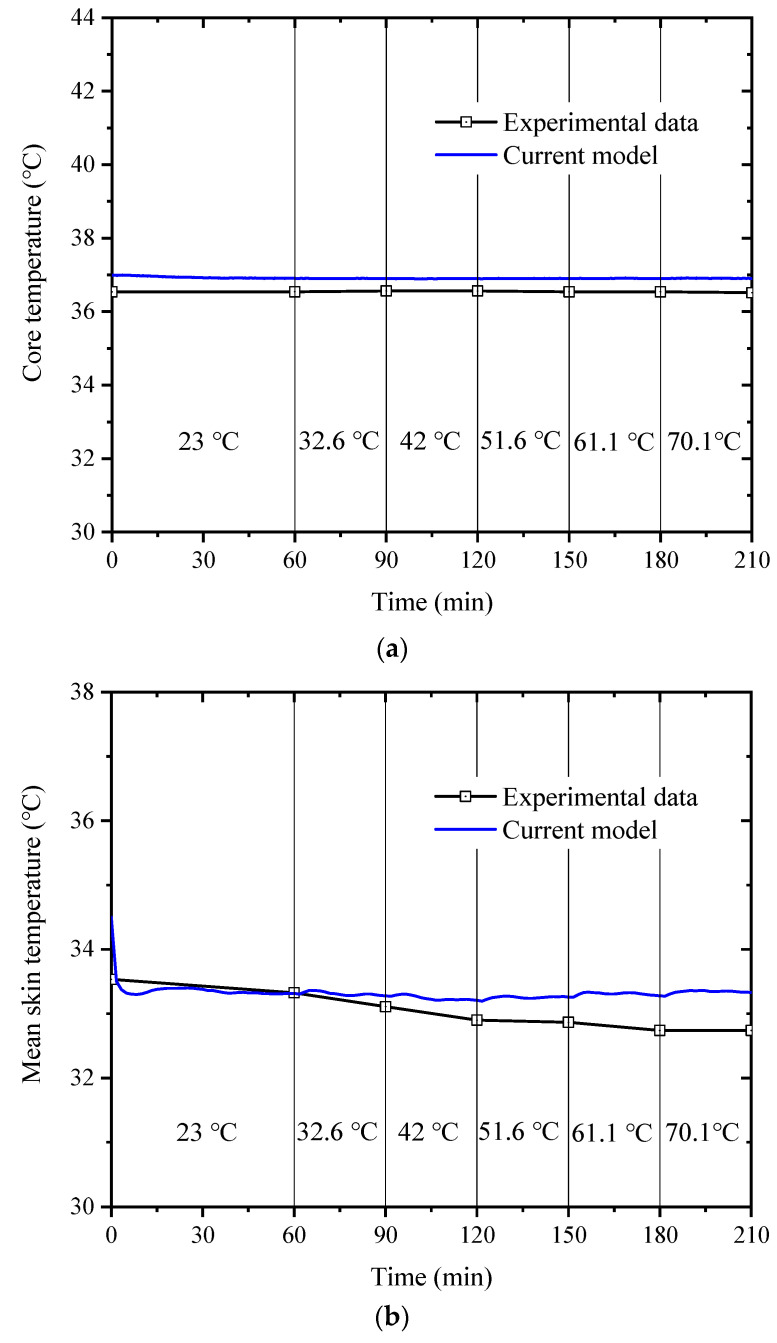
Comparison of core temperature (**a**) and mean skin temperature (**b**) between the experimental data [38] and the simulated results calculated with the current model.

Concerning the core temperature (a) and mean skin temperature (b), the comparisons of the experimental data [38] with the simulation results of the current model are shown in Figure 4. As a result, the model-predicted core temperature and mean skin temperature differed most from the experimental data by 0.45 °C and 0.96 °C, respectively. Therefore, the modified model can accurately predict the core and average skin temperature throughout the process.

**Figure 5:**
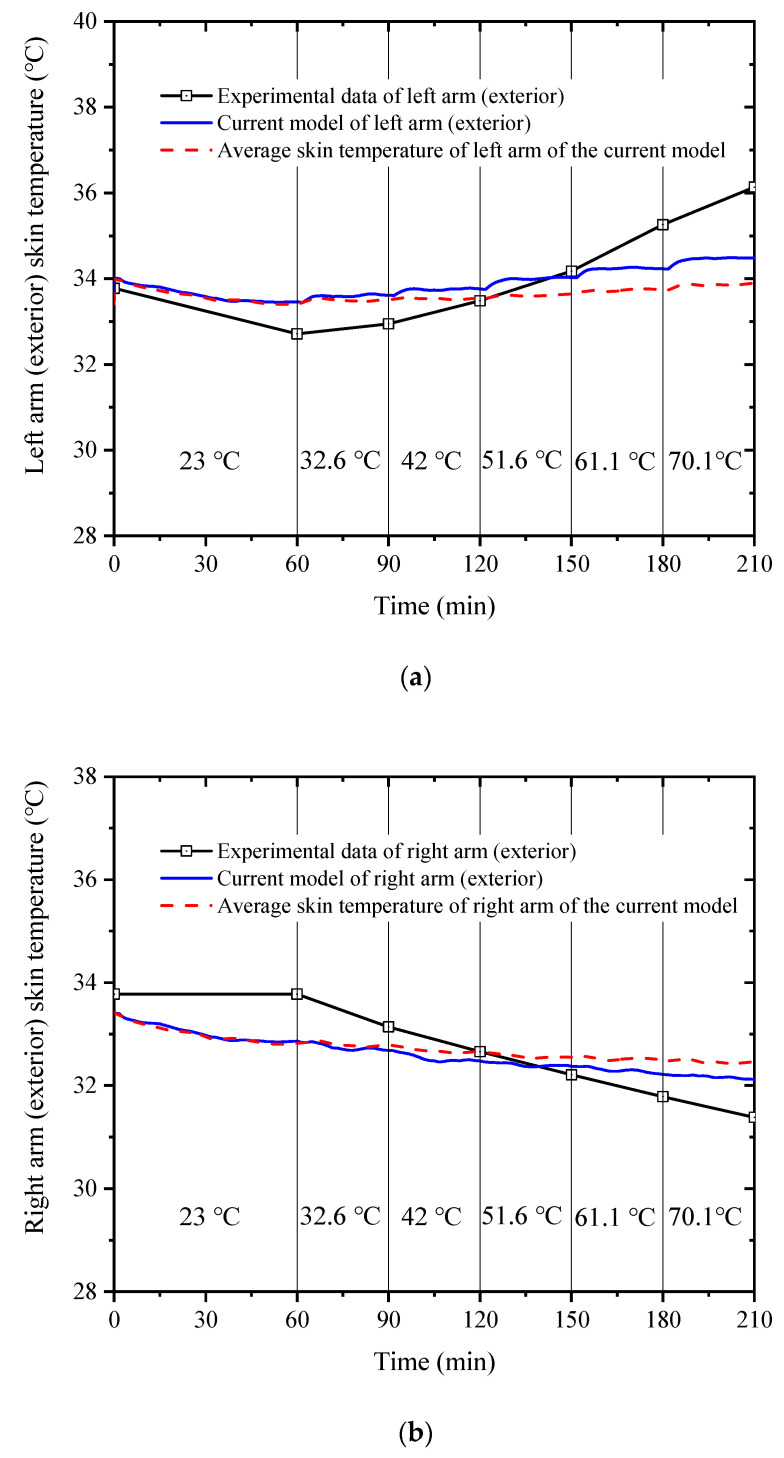

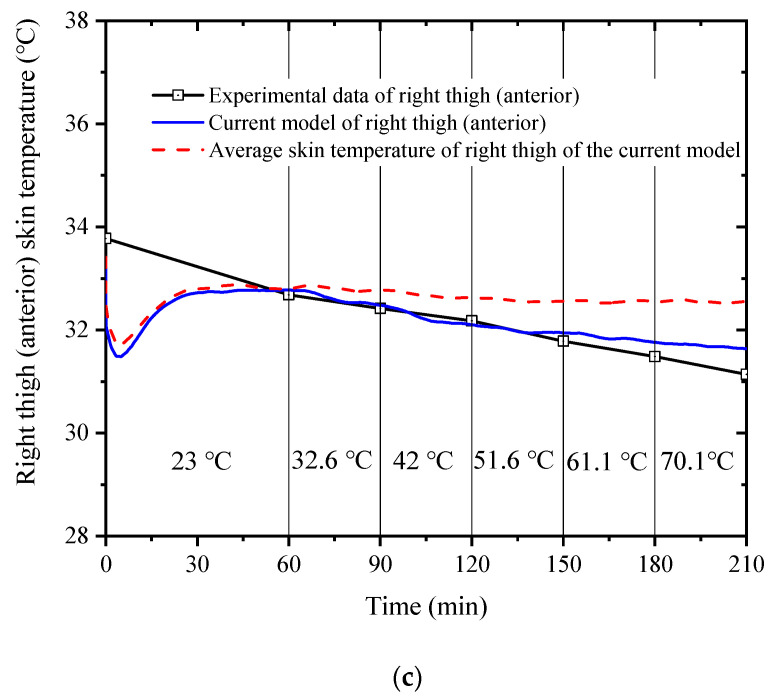
Comparison of the left arm (exterior) skin temperature (**a**), right arm (exterior) skin temperature, (**b**) and right thigh (anterior) skin temperature (**c**) between the experimental data [38] and the simulated results calculated with the current model.

Figure 5 shows the comparison of the left arm (exterior) skin temperature (a), right arm (exterior) skin temperature (b), and right thigh (anterior) skin temperature (c) between the experimental data [38] and the simulated results calculated with the current model. The calculated temperatures of the three body segments are in good agreement with the experimental data, and the maximum difference is within 2 °C. The results show that the extended model can accurately predict the human core temperature and skin temperature in asymmetric high-temperature environments.

Additionally, the unilateral skin temperature of the concerned body segment is more accurate compared to its average value from Figure 5. The reason is that the spatial temperature distribution is asymmetric, and the ambient radiation temperature on the side close to the heat source is higher, resulting in a higher risk of local heat stress and local heat discomfort in the corresponding human body segment. Conversely, on the side away from the heat source, the ambient radiant temperature is lower, resulting in a lower risk of localized heat stress on the corresponding body part. This means that previous models, which treat each part of the body as a uniform column, are not suitable for non-uniform high-temperature environments. Therefore, it is necessary to consider the sensitivity of modeling to changes in skin temperature angles when predicting the risk of local heat stress in workers in a non-uniform high-temperature environment.

## 4. Conclusions

In this paper, a multi-segment human bioheat model was first extended to the asymmetric environment by considering the sensitivity of the modeling to angular changes in skin temperature. The human body was subdivided into 15 cylindrical segments, each containing a core node, a vein node, an artery node, and four skin nodes, for a total of 105 nodes. Then, the effects of high temperatures on metabolic rate, the cardiovascular system, and the role of heat-induced dehydration on sweating and skin vasodilation were considered to further extend this model for human thermal response prediction in asymmetric high-temperature environments.

The comparison of predicted results from previous models and experimental data indicate that the extended model can accurately simulate core, average skin, and local skin temperatures under both uniform and non-uniform high-temperature conditions. The analysis of the results revealed that the extended model could predict the human skin temperature more accurately in uniform high-temperature environments. Under non-uniform high-temperature conditions, the local skin temperature predictions agreed better with the experimental data, with the maximum difference within 2 °C. Therefore, the model can be used as an effective tool to estimate human physiological and thermoregulatory responses under uniform and non-uniform high-temperature environments.

In conclusion, this paper established a thermophysiological response model for workers in a non-uniform high-temperature environment. The model can accurately calculate the heat transfer process between the non-uniform heat source and the human based on the actual scene between them. It can not only be applied to fire scenes, steel grid work environments, and architectural scenes, but also to life scenes with local heat sources, such as bathroom bathing and indoor heating. The application of the modified model is extended by taking into account specific human thermal responses and physiological equations in extremely high-temperature environments. The output parameter of the model is the local skin temperature, which supports the calculation of local thermal stress and local thermal discomfort. In particular, it can predict parameters such as localized thermal burns, protecting operators at high temperatures. It will also provide the improvement suggestions of protective clothing to avoid irreparable burns or life-threatening damage.

Future work will verify the modified model through experiments, adding a refined high-temperature protective clothing model, especially a cooling system.

## Figures and Tables

**Table 1:** The detailed parameters of each segment used in the model [11,13,17].

Body Segments	*A* (m^2^)	*M_cr_* (W)	*M_sk_* (W)	*C_cr_* (J/K)	*C_bl,a_* (J/K)	*C_bl,v_* (J/K)	*C_sk_* (J/K)	*K_cr-sk_* (W/K)	*m_sk,basal_* (cm^3^/h)	*m_sk,min_* (cm^3^/h)	*m_sk,max_* (cm^3^/h)
Head	0.140	18.433	0.219	122,47.1	810.5	2482.1	1710.2	1.848	6050	4518	16,552
Upper arm	0.096	1.062	0.182	6707.9	49.6	151.9	1284.02	1.304	910	0	8319
Forearm	0.063	0.593	0.101	3750.1	32.3	99.1	717.9	0.913	508	0	5553
Hand	0.050	0.095	0.093	791.4	21.4	65.7	723.5	0.545	1114	627	4454
Chest	0.336	5.95	0.6	36,732.1	2266.8	6941.9	13,624.8	4.921	3441.5	0	33,246
Abdomen	0.221	46.86	0.6	67,160.5	566.7	1735.5	13,624.8	4.921	2272.5	0	21,953
Thigh	0.209	1.708	0.343	11,926.3	93.1	285.1	4133.9	1.564	1456	0	12,453
Calf	0.112	0.754	0.153	5262.3	36.8	112.6	1850.8	1.216	651	0	8253
Foot	0.056	0.146	0.127	1465.3	18.7	57.2	1347.4	0.631	934	301	5278

**Table 2:** Distribution coefficients of each body segment [11].

Body Segments	*T_sk_*_0_ (°C)	Distribution Coefficients		
		*α* *_er_*	*α* *_sh_*	*α* *_sw_*
Head	35.6	0.07	0.02	0.081
Chest	33.6	0.281	0.485	0.275
Abdomen	33.3	0.212	0.365	0.206
Left and right upper arms	33.4	0.023	0.004	0.051
Left and right forearms	34.6	0.012	0.026	0.026
Left and right hands	35.2	0.092	0	0.016
Left and right thighs	33.8	0.05	0.023	0.073
Left and right calves	33.4	0.025	0.012	0.036
Left and right feet	33.9	0.017	0	0.018

**Table 3:** Relative error between the predicted results of the two models and the experimental data.

	Stage 1		Stage 2		Stage 3		Stage 4	
	Current Model	Yang et al. [18] Model	Current Model	Yang et al. [18] Model	Current Model	Yang et al. [18] Model	Current Model	Yang et al. [18] Model
Core temperature	±0.40%	±0.14%	±0.27%	±0.27%	±0.33%	±0.24%	±0.47%	±0.29%
Mean skin temperature	±0.41%	±0.97%	±0.67%	±0.79%	±1.62%	±1.50%	±0.30%	±1.27%

## Data Availability

Not applicable.

## Nomenclature

*A*	area (m^2^)
*C*	thermal capacitance (J/K)
*c*	specific heat
*Clds*	integrated cold signals (°C)
*CON*	the heat exchange caused by convection (W)
*D*	the cumulative water loss from the human body (%)
*EVA*	the heat exchange caused by evaporation (W)
*HR*	heart rate (beat/min)
*F_vf_*	the veiw factor
*h_a_*	convection coefficient of the blood in the artery (W/m^2^K)
*h_c_*	heat convection coefficient (W/m^2^K)
*h_e_*	heat evaporation coefficient (W/m^2^Pa)
*h_r_*	heat radiation coefficient (W/m^2^K)
*h_v_*	convection coefficient of the blood in the vein (W/m^2^K)
*K*	conductance (W/K)
L	the segmental length (m)
*M*	segmental metabolic rate (W)
*M*_0_	thermoneutrality metabolic rate (W)
$\dot{m}$	mass flow rate (kg/s)
*P*	vapor pressure (Pa)
*Q*	heat transfer (W)
*RAD*	the heat exchange caused by radiation (W)
*RES*	respiratory heat loss (W)
*r*	the radius of the skin layer (m)
*T*	temperature (°C)
*t*	time (s)
*th*	the thickness of the skin layer in the segment (m)
*W*	mechanical work (W)
*Wrms*	integrated warm signals (°C)
*Subscript*	
*a*	artery
*a,adj*	adjacent element of artery blood
*air*	ambient air
*bl*	blood
*bl,a*	artery blood
*bl,a,adj*	adjacent element of artery blood
*bl,v*	vein blood
*bl,v,adj*	adjacent element of vein blood
*cr*	core
*cr-a*	exchange between core and artery
*cr-sk*	exchange between core and skin
*cr-v*	exchange between core and vein
*i*	skin node sector index (i = 1–4)
*mrt*	mean radiant temperature
*max*	maximum
*min*	minimum
*n*	serial number of different nodes of each body part
*perfusion, a-cr*	blood perfusion from artery to core
*perfusion, cr-v*	blood perfusion from core to vein
*perfusion, cr-sk*	blood perfusion between core and skin
*sh*	shiver
*sk*	skin
*sw*	sweat
*v*	vein
*v,adj*	adjacent element of vein blood
Greek symbols	
α	distribution coefficient
θ	the corresponding angular coordinate of the skin node

## References

[1] Charney C.K. (1992). Mathematical models of bioheat transfer. Adv. Heat Transf..

[2] Xu X., Tikuisis P. (2014). Thermoregulatory modeling for cold stress. Compr. Physiol..

[3] Pennes H.H. (1948). Analysis of tissue and arterial blood temperature in the resting human forearm. J. Appl. Phys..

[4] Weinbaum S., Jiji L. (1984). Theory and experiment for the effect of vascular temperature on surface tissue heat transfer—Part 1: Anatomical foundation and model conceptualization. J. Biomech. Eng.-Trans. Asme.

[5] Gagge A.P., Nishi Y. (1971). An effective temperature scale based on a simple model of human physiological regulatory response. ASHRAE Trans..

[6] Stolwijk J.A.J., Charles C. (1970). Mathematical model of thermoregulation. Physiological and Behavioral Temperature Regulation.

[7] Stolwijk J.A.J. (1971). A Mathematical Model of Physiological Temperature Regulation in Man.

[8] Wissler E.H., Shitzer A., Eberhardt R.C. (1985). Mathematical simulation of human thermal behavior using whole body models. Heat Mass Transfer in Medical Biology.

[9] Huizenga C., Hui Z. (2001). A model of human physiology and comfort for assessing complex thermal environments. Buiding Environ..

[10] Fiala D., Lomas K.J., Stohrer M. (1999). A computer model of human thermoregulation for a wide range of environmental conditions: The passive system. J. Appl. Physiol..

[11] Tanabe S., Kobayashi K., Nakano J., Ozeki Y., Konishi M. (2002). Evaluation of thermal comfort using combined multi-node thermoregulation (65MN) and radiation models and computational fluid dynamics (CFD). Energy Build..

[12] Kobayashi Y., Tanabe S. (2013). Development of JOS-2 human thermoregulation model with detailed vascular system. Build. Environ..

[13] Salloum M., Ghaddar N., Ghali K. (2007). A new transient bioheat model of the human body and its integration to clothing models. Int. J. Therm. Sci..

[14] Ferreira M.S., Yanagihara J.I. (2009). A transient three-dimensional heat transfer model of the human body. Int. Commun. Heat Mass Transf..

[15] Zolfaghari A., Maerefat M. (2010). A new simplified thermoregulatory bioheat model for evaluating thermal response of the human body to transient environments. Build. Environ..

[16] Karaki W., Ghaddar N., Ghali K., Kuklane K., Holmer I., Vanggaard L. (2013). Human thermal response with improved AVA modeling of the digits. Int. J. Therm. Sci..

[17] Weng W.G., Han X.F., Fu M. (2014). An extended multi-segmented human bioheat model for high temperature environments. Int. J. Heat Mass Transf..

[18] Yang J., Weng W., Zhang B. (2014). Experimental and numerica lstudy of physiological responses in hot environments. J. Therm. Biol..

[19] Xie Y., Niu J., Zhang H. (2020). Development of a multi-nodal thermal regulation and comfort model for the outdoor environment assessment. Build. Environ..

[20] Takahashi Y., Nomoto A., Yoda S., Hisayama R., Ogata M., Ozeki Y., Tanabe S. (2021). Thermoregulation model JOS-3 with new open source code. Energy Build..

[21] Itani M., Ghaddar N., Ghali K., Laouadi A. (2020). Bioheat modeling of elderly and young for prediction of physiological and thermal responses in heat-stressful conditions. J. Therm. Biol..

[22] Wu J.S., Hu Z.Q., Gu Y., Li L.T., Zhu H.Z. (2022). A multi-segmented human bioheat model for cold and extremely cold exposures. Int. J. Therm. Sci..

[23] Huizenga C., Hui Z., Arens E., Wang D. (2004). Skin and core temperature response to partial- and whole-body heating and cooling. J. Therm. Biol..

[24] Conceio E.Z.E., Lúcio M.M.J.R., Loureno T.M.C., Brito A.I.P.V. (2006). Evaluation of Thermal Comfort in Slightly Warm Ventilated Spaces in Nonuniform Environments. HVAC&R Res..

[25] Iyoho A., Jang T., Nair S. Human thermal model with extremities for asymmetric environments. Proceedings of the 2004 American Control Conference.

[26] Al-Othmani M., Ghaddar N., Ghali K. (2008). A multi-segmented human bioheat model for transient and asymmetric radiative environments. Int. J. Heat Mass Transf..

[27] Fiala D., Psikuta A., Jendritzky G. (2010). Physiological modeling for technical, clinical and research applications. Front. Biosci..

[28] Zhang H., Arens E., Huizenga C., Han T. (2010). Thermal sensation and comfort models for non-uniform and transient environments, part III: Whole-body sensation and comfort. Build. Environ..

[29] Zhang H., Arens E., Huizenga C., Han T. (2010). Thermal sensation and comfort models for non-uniform and transient environments: Part I: Local sensation of individual body parts. Build. Environ..

[30] Lai D., Chen Q. (2016). A two-dimensional model for calculating heat transfer in the humanbody in a transient and non-uniform thermal environment. Energy Build..

[31] Wang Y., Lian Z. (2018). A thermal comfort model for the non-uniform thermal environments. Energy Build..

[32] Han X.F. (2012). Experimental and Numerical Study of Human Thermal Response in High-Temperature Environment. Ph.D. Thesis.

[33] Zhao J., Wang H., Li Y. (2020). Heatstroke recovery at home as predicted by human thermoregulation modeling. Build. Environ..

[34] Kubaha K. (2005). Asymmetric Radiant Fields and Human Thermal Comfort. Ph.D. Thesis.

[35] (2001). ASHRAE Handbook of Fundamentals.

[36] Wenzel U.D. (1962). Pelztiergesundheitsdienst.

[37] Montain S.J., Latzka W.A., Sawka M.N. (1995). Control of thermoregulatory sweating is altered by hydration level and exercise intensity. J. Appl. Physiol..

[38] Fanger P.O., Ipsen B.M., Langkilde G., Olessen B.W., Christensen N.K., Tanabe S. (1985). Comfort limits for asymmetric thermal radiation. Energy Build..

